# 肺癌合并其他器官多原发癌的临床特点分析

**DOI:** 10.3779/j.issn.1009-3419.2021.101.01

**Published:** 2021-01-20

**Authors:** 帅 张, 志云 许, 高超 董, 明 李, 林 许

**Affiliations:** 210009 南京，江苏省肿瘤医院 & 江苏省肿瘤防治研究所 & 南京医科大学附属肿瘤医院胸外科；江苏省恶性肿瘤分子生物学及转化医学重点实验室 Department of Thoracic Surgery, Jiangsu Cancer Hospital & Jiangsu Institute of Cancer Research & The Affiliated Cancer Hospital of Nanjing Medical University; Jiangsu Key Laboratory of Molecular and Translational Cancer Research, Nanjing 210009, China

**Keywords:** 肺肿瘤, 多原发癌, 同时多原发癌, 异时多原发癌, Lung neoplasms, Multiple primary malignancies, Synchronous multiple primary malignancies, Metachronous multiple primary malignancies

## Abstract

**背景和目的:**

随着胸部计算机断层扫描（computed tomography, CT）肺癌早筛技术的普及，肺癌合并其他器官多原发癌（multiple primary malignancies, MPM）的检出比例不断提高。本研究就发病率、病理特征、诊疗特点和预后情况进行探讨分析，为完善此类疾病的临床诊疗提供研究依据。

**方法:**

2011年9月-2015年9月江苏省肿瘤医院胸外科共收治5, 570例肺癌患者，回顾性分析其中61例肺癌合并其他器官MPM患者的临床病理特征。

**结果:**

本研究中肺癌合并其他器官MPM的发病率为1.1%，其中同时多原发癌（synchronous MPM, SMPM）15例，异时多原发癌（metachronous MPM, MMPM）46例；结直肠癌、乳腺癌和甲状腺癌占肺癌合并其他器官MPM的前三位；患者总体5年生存率为39.3%，有71.4%的患者死于肺癌转移或复发，多因素分析发现肺癌患者的临床分期、肺癌与其他肿瘤发生的先后顺序、合并肿瘤的治疗状态以及是否存在表皮生长因子受体（epidermal growth factor receptor, *EGFR*）基因突变是影响患者生存期的重要因素。

**结论:**

肺癌合并其他器官MPM的发病率并非罕见，相较于其他器官恶性肿瘤，肺癌是主要致死原因，中晚期肺癌、SMPM、肺癌先发、合并肿瘤仅获姑息治疗和无*EGFR*基因突变的患者预后较差。

## Abstract (en)

**Background and objective:**

With the popularization of chest computed tomography (CT) early screening for lung cancer, the detection rate of lung cancer combined with multiple primary malignancies (MPM) in other organs has been increasing. In this paper, the incidence, pathological characteristics, diagnosis and treatment characteristics and prognosis were discussed and analyzed to provide research basis for improving the clinical diagnosis and treatment of this disease.

**Methods:**

From September 2011 to September 2015, a total of 5, 570 patients with lung cancer were treated in Jiangsu Cancer Hospital. The clinicopathological characteristics of 61 patients with lung cancer combined with MPM in other organs were retrospectively analyzed.

**Results:**

The incidence rate of lung cancer combined with MPM in other organs in this group was 1.1%, of which 15 cases were synchronous MPM (SMPM), 46 cases were metachronous MPM (MMPM). Colorectal cancer, breast cancer and thyroid cancer accounted for the top three of lung cancer combined with MPM in other organs. The overall 5-year survival rate was 39.3% and 71.4% of patients died from metastasis or recurrence of lung cancer. Multivariate analysis showed that the clinical stage of lung cancer patients, the order of occurrence of lung cancer and other tumors, the treatment status of patients with other organ tumors and the presence of epidermal growth factor receptor (*EGFR*) gene mutation were important factors for the survival of the patients.

**Conclusion:**

The incidence rate of lung cancer combined with MPM in other organs is not uncommon. Lung cancer is the main cause of death compared with other organs tumors. Patients with advanced lung cancer, SMPM, lung cancer first, combined with tumor only receiving palliative treatment and without *EGFR* gene mutation had a poor prognosis.

近年来，胸部计算机断层扫描（computed tomography, CT）肺癌早筛工作的普及提高了早期肺癌在总体样本中的构成比^[1]^，靶向药物的应用显著延长了部分驱动基因突变敏感性肺癌患者的生存期^[2]^。这部分肺癌患者生存期足够长，以至于随访观察中合并其他器官原发恶性肿瘤的比例也显著提高。这种同一患者先后或同时发生2个或2个以上的原发恶性肿瘤的情况称之为多原发癌（multiple primary malignancies, MPM），按照发生的时序性可分为同时多原发癌（synchronous MPM, SMPM）和异时多原发癌（metachronous MPM, MMPM）^[3]^。本研究回顾性分析了江苏省肿瘤医院胸外科2011年9月-2015年9月收治的肺癌合并其他器官MPM的临床特点。

## 1 资料与方法

### 1.1 一般资料

2011年9月-2015年9月江苏省肿瘤医院胸外科共收治5, 570例肺癌患者，对其中合并其他器官MPM（诊断参照Warren和Gates标准^[4]^）的患者进行临床和病理资料的回顾性分析。本组MPM的诊断尤其注意排除其他器官原发恶性肿瘤转移至肺的情况，在此基础上将MMPM根据其他器官原发恶性肿瘤与肺癌诊断的前后顺序分为肺癌先发组（lung cancer first, LCF）和其他器官先发组（other cancer first, OCF）。患者的临床资料包括年龄、性别、肺癌的位置、分期、肺癌组织类型、数目、发生时间、治疗方法和生存情况等。随访结果来自定期门诊复查、病案室及电话或信件联系等方式获得的随访资料。患者第一原发肿瘤确诊日至死亡或随访结束日计为生存期，以月为单位。

### 1.2 统计学方法

数据分析采用SPSS 21.0统计软件。临床病理特点的比较采用独立样本*t*检验和卡方检验，采用*Kaplan-Meier*法利用Graph pad Prism 8软件绘制生存曲线。单因素分析应用*Log-rank*检验，多因素分析采用*Cox*风险回归模型，*P* < 0.05被认为有统计学差异。

## 2 结果

### 2.1 一般资料

本组共有肺癌合并其他器官MPM的患者61例，占同期收治的5, 570例肺癌患者的1.1%，SMPM和MMPM组分别占15例和46例，MMPM组中LCF患者数量明显少于OCF组（表 1）。初次就诊时有明显咳嗽、咯血、胸痛等典型临床表现的患者有14例，其余均为随访复查或健康体检时发现。吸烟史和组织病理学类型在SMPM和MMPM两组之间差异具有统计学意义（*P* < 0.05），其余指标分布差异无统计学意义（*P* > 0.05）。

**表 1:** 61例肺癌合并其他器官恶性肿瘤患者的临床资料 Clinical characteristics of the 61 lung cancer patients with accompanying malignancies

Characteristics	Total (*n*=61)	Synchronous group (*n*=15)	Metachronous group (*n*=46)	*P*
Age (yr)				0.239
< 65	36	7	29	
≥65	25	8	17	
Gender				0.165
Male	21	8	13	
Female	40	7	33	
Family history of cancer				0.368
Yes	12	3	9	
No	49	12	37	
Smoking history				< 0.001
Non-smoking	48	10	38	
Smoking	13	5	8	
Histological type				0.072
Adenocarcinoma	49	11	38	
Squamous cell carcinoma	9	3	6	
Others	3	1	2	
Stage for lung cancer				0.063
Ⅰ	25	5	20	
Ⅱ	20	3	17	
Ⅲ	16	7	9	
Operation selection				0.140
Video-assisted thoracic surgery	50	11	39	
Traditional thoracotomy	11	4	7	
Order of occurrence				
LCF			15	
OCF			31	

### 2.2 肺癌合并其他器官MPM的分布状况

61例患者中，仅有2例是三原发癌，其余59例为双原发癌。结直肠癌、乳腺癌和甲状腺癌分列肺癌合并其他器官MPM的前三位。本组MPM合并肺外器官肿瘤的分布和个数见表 2。

**表 2:** 61例患者合并肺外肿瘤的分布情况 Distribution of 61 patients with malignancies accompanying lung cancer

Tumor	Total (*n*=63^*^)	Synchronous group (*n*=15)	Metachronous group (*n*=48^*^)	LCF group (*n*=14)	OCF group (*n*=32^*^)
Colonrectal cancer	14	1	13	2	11
Breast cancer	12	4	8	3	5
Thyroid carcinoma	11	3	8	4	4
Esphagus cancer	6	3	3	1	2
Cervical carcinoma	3	0	3	0	3
Thymic carcinoma	2	1	1	0	1
Larynx cancer	2	0	2	1	1
Renal carcinoma	2	1	1	0	1
Bladder carcinoma	2	0	2	1	1
Gastric cancer	2	1	1	0	1
Laryngocarcinoma	2	0	2	1	1
Hematological tumors	2	1	1	0	1
Skin cancer	1	0	1	1	0
Tongue cancer	1	0	1	0	1
Testicular cancer	1	0	1	0	1
^*^There are two patients with triple primary cancers.					

### 2.3 随访与预后

所有病例随访至2020年9月，期间有3例失访，无围手术期死亡病例。患者总体5年生存率为39.7%，71.4%的死亡病例是因肺癌转移或复发所致，SMPM组预后明显差于MMPM组，中位生存时间分别为35个月和55个月。在MMPM组中，LOF组预后稍差于OCF组，中位生存时间分别为55个月和57个月（图 1）。

**图 1:**
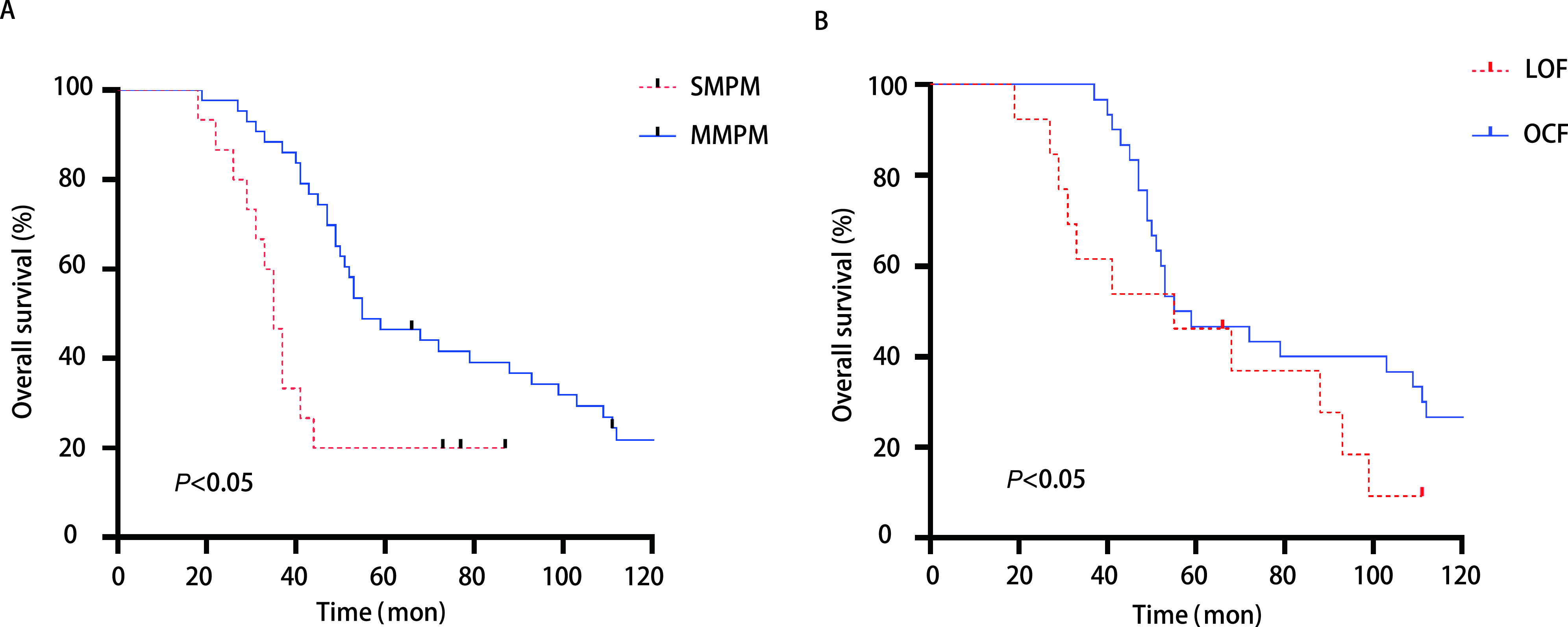
肺癌合并其他器官MPM患者的生存期分析。A：MMPM组预后明显好于SMPM组，中位生存时间分别为55个月和35个月（*P* < 0.05）；B：在MMPM组中，OCF组预后稍好于LOF组，中位生存时间分别为57个月和55个月（*P* < 0.05）。 Survival analysis of patients with lung cancer combined with MPM of other organs. A: MMPM patients demonstrated significant better OS than SMPM patients, with median OS of 55 mon and 35 mon, respectively (*P* < 0.05); B: OCF patients demonstrated better OS than LOF patients, with median OS of 57 mon and 55 mon, respectively (*P* < 0.05). MPM: multiple primary malignancies; SMPM: synchronous MPM; MMPM: metachronous MPM; LCF: lung cancer first; OCF: other cancer first; OS: overall survival.

### 2.4 预后影响因素分析

对患者预后进行单因素分析，发现肺癌患者的年龄和吸烟史是预后影响因素，但多因素分析显示差异无统计学意义（*P* > 0.05）。单因素和多因素分析均证实肺癌的临床病理分期、肿瘤发生的时序性、肺癌手术方式的选择、其他器官肿瘤的治疗状态以及表皮生长因子受体（epidermal growth factor receptor, *EGFR*）基因突变状态是影响患者预后的重要因素（表 3），即为中晚期肺癌、SMPM、肺癌先发和无*EGFR*基因突变的患者预后差，接受胸腔镜手术治疗的患者预后优于传统开胸，其他器官肿瘤接受姑息治疗的患者预后差，反之预后更好。

**表 3:** 影像MPM患者预后的单因素和多因素分析结果 Univariate and multivariate survival analysis of prognostic factors in MPM patients

Characteristics	5-yr OS rate (%)	Univariate analysis			Multivariate analysis	
		HR(95%CI)	*P*		HR(95%CI)	*P*
Age (yr)		0.094 (0.031-0.283)	< 0.001		0.320 (0.059-1.743)	0.188
< 65	40.0					
≥65	39.1					
Gender		0.594 (0.295-1.196)	0.145		0.421 (0.193-1.862)	0.443
Male	30.0					
Female	44.7					
Smoking history		2.186 (1.004-4.759)	0.049		1.694 (0.671-4.274)	0.265
Non-smoking	41.7					
Smoking	30.0					
Histological type		1.585 (0.649-3.970)	0.312		1.392 (0.463-5.180)	0.739
Adenocarcinoma	35.4					
Squamous cell carcinoma	28.6					
Others	66.7					
Stage for lung cancer		4.986 (2.916-8.527)	< 0.001		4.427 (2.456-7.979)	< 0.001
Ⅰ	70.8					
Ⅱ	26.7					
Ⅲ	5.3					
*EGFR* mutation		0.461 (0.223-0.954)	0.037		0.385 (0.146-1.016)	0.045
Mutant	46.4					
Wild-type	33.3					
Operation selection		1.367 (0.793-3.021)	< 0.001		1.781 (1.004-2.157)	< 0.001
Video-assisted thoracic surgery	43.8					
Traditional thoracotomy	20.0					
Other cancers status		3.932 (1.786-8.657)	0.001		3.484 (1.458-8.321)	0.005
Curative	45.5					
Palliative	21.4					
Time of occurrence		0.243 (0.112-0.524)	< 0.001		0.267 (0.083-0.865)	0.028
Synchronous	20.0					
Metachronous	46.5					
Order of occurrence		0.461 (0.216-0.988)	0.046		0.751 (0.170-3.314)	0.016
LCF	23.1					
OCF	44.4					

## 3 讨论

目前，我国肺癌发病率仍在逐年升高，随着胸部CT检查的普及，虽然早期肺癌的检出率明显升高，但肺癌的总体预后依然不佳，高居癌症死因的首位^[5]^。合并其他原发肿瘤的肺癌患者占肺癌总体人群比例较低，目前国内相关报道较少，本研究中肺癌合并其他器官MPM的患者占同期肺癌总病例的1.1%，由于本研究入组对象是江苏省肿瘤医院胸外科收治的手术治疗患者，对于无法进行手术治疗的患者，如晚期肺癌患者，均不纳入本研究，故发病率稍低于既往的一些报道^[6-8]^。随着我国肺癌早筛工作的逐渐普及，合并肺癌的MPM发病率很可能会进一步提高。

本组中肺腺癌的比例明显高于肺鳞癌，符合目前肺腺癌的流行病学特点。与其他研究^[3]^不同的是，本组病例合并的其他恶性肿瘤没有表现出明显的组织器官特异性，全身多部位肿瘤均有合并存在的可能性。其中，结直肠癌、乳腺癌和甲状腺癌占肺癌合并其他器官原发恶性肿瘤的前三位，作者推测这三种肿瘤均为常见肿瘤，伴发肺癌的比例也相应较高。目前，MPM发生机理尚不明确，认为可能的机理主要包括三类^[6]^：遗传因素、免疫系统和在治疗过程中是否经历过放化疗。本研究中具有明显家族肿瘤遗传史的患者比例较低，但是有约一半的患者发生第一原发癌后都曾接受过足量的化疗和（或）放疗，从发病机理推测既往的放化疗诱发机体DNA突变和免疫损伤导致第二或第三原发癌。

本组研究中单因素和多因素分析均支持肺癌的临床病理分期、肿瘤发生的时序性、肺癌手术方式的选择、其他器官肿瘤的治疗状态以及*EGFR*基因突变状态是影响患者预后的重要因素。肺癌的临床分期和合并其他器官肿瘤的治疗状态影响MPM的预后，这一结论符合一般常识性理解。肿瘤发生的时序性影响患者预后的研究结果与既往的文献^[3, 6, 9]^类似，本研究中SMPM组与MMPM组的生存曲线具有较好的区分度，中位生存时间分别为35个月和55个月。研究^[10]^表明，MMPM的患者相对于SMPM的预后较好，原因可能是机体同时或较短时间内接受多重抗肿瘤治疗，使得免疫系统不能及时修复，减弱了机体抗肿瘤免疫的功能。另一方面，MMPM患者在经历第一原发癌的诊疗后会进行较长时间的随访复查，在此过程中，第二原发癌更容易经早期筛查发现，从而达到更好的治疗效果。

肺腺癌相较于肺鳞癌具有更多的*EGFR*突变可能^[11]^，本研究中肺腺癌的比例明显高于肺鳞癌，*EGFR*基因的突变比例是48.3%，其中有21例患者在治疗期间曾口服靶向药物，这部分患者的生存期相对于同分期的肺鳞癌和无突变的肺腺癌患者得到了明显延长。既往已有研究^[12]^表明吸烟是MPM的危险因素，本研究发现吸烟仅在单因素分析中对预后有影响，其中吸烟人群主要集中于肺鳞癌的患者，此类患者伴发头颈部鳞癌的比例高于未吸烟患者，且预后较未吸烟患者明显更差。

MMPM的治疗模式和单原发癌相同，SMPM应根据具体情况同期或分期完成治疗。MPM需排除转移和复发的可能，因为两者的治疗方法和预后有很大差异。一般情况下，MPM患者预后优于单原发癌复发和转移的患者。肺癌合并其他器官MPM治疗后的5年生存率为14.0%-66.1%不等，多数报道肺癌仍是最主要的死因。本组患者的总体5年生存率为39.7%，肺癌是主要死因，与相关文献报道^[3, 6, 13]^的结果类似，纳入研究的患者以外科手术为主，术后或转移复发后采用放化疗或靶向药物治疗。手术方式的选择是影响患者预后的重要因素，这一结果在既往文献中未见相关报道。手术方式影响预后的原因可能由于其方案制定具有一定的病情偏倚，外科医生对于复杂的、病情偏晚的患者更倾向于选择传统开胸手术，而对于外周型、早中期的患者更倾向于选择胸腔镜微创手术。

最后，需要特别指出的是，因为有半数以上的异时癌发生在肺癌诊断前后的5年以外的时间，所以第一原发癌无疾病生存期超过5年也不能放弃长期随访，在诊疗过程中应注意严格区别是第二原发癌还是第一原发癌的复发或转移，以便能够早诊早筛异时癌，提高治疗效果。
